# Age of second language acquisition in multilinguals has an impact on gray matter volume in language-associated brain areas

**DOI:** 10.3389/fpsyg.2015.00638

**Published:** 2015-06-08

**Authors:** Anelis Kaiser, Leila S. Eppenberger, Renata Smieskova, Stefan Borgwardt, Esther Kuenzli, Ernst-Wilhelm Radue, Cordula Nitsch, Kerstin Bendfeldt

**Affiliations:** ^1^Department of Social Psychology and Social Neuroscience, Institute of Psychology, University of Bern, BernSwitzerland; ^2^Medical Image Analysis Centre, University Hospital Basel, BaselSwitzerland; ^3^Department of Psychiatry, University Hospital Basel, University of Basel, BaselSwitzerland; ^4^Division of Infectious Diseases and Hospital Epidemiology, University Hospital Basel, BaselSwitzerland; ^5^Department of Biomedicine, Institute of Anatomy, University of Basel, BaselSwitzerland

**Keywords:** multilingualism, bilingualism, age of L2 acquisition, magnetic resonance imaging, gray matter volume

## Abstract

Numerous structural studies have established that experience shapes and reshapes the brain throughout a lifetime. The impact of early development, however, is still a matter of debate. Further clues may come from studying multilinguals who acquired their second language at different ages. We investigated adult multilinguals who spoke three languages fluently, where the third language was learned in classroom settings, not before the age of 9 years. Multilinguals exposed to two languages simultaneously from birth (SiM) were contrasted with multinguals who acquired their first two languages successively (SuM). Whole brain voxel based morphometry revealed that, relative to SuM, SiM have significantly lower gray matter volume in several language-associated cortical areas in both hemispheres: bilaterally in medial and inferior frontal gyrus, in the right medial temporal gyrus and inferior posterior parietal gyrus, as well as in the left inferior temporal gyrus. Thus, as shown by others, successive language learning increases the volume of language-associated cortical areas. In brains exposed early on and simultaneously to more than one language, however, learning of additional languages seems to have less impact. We conclude that – at least with respect to language acquisition – early developmental influences are maintained and have an effect on experience-dependent plasticity well into adulthood.

## Introduction

In recent years, numerous studies on neuronal plasticity have established that training results in structural changes in critically involved cortical brain areas. On a macroscopic level, it has been shown that gray matter (GM) density and GM volume are altered after different kinds of training (for review see Taubert et al., 2010 and Zatorre et al., 2012). Within the domain of neurolinguistics too, ongoing research has demonstrated that acquiring a second language (L2) has a substantial influence on the anatomy of the brain (Li et al., 2014; Stein et al., 2014). This was the case for a variety of language characteristics, such as non-native speech sounds (Golestani et al., 2002, 2007, 2011; Crinion et al., 2006; Wong et al., 2008), acquisition of vocabulary (Grogan et al., 2009, 2012; Hosoda et al., 2013), reading skills (Cummine and Boliek, 2013; Zhang et al., 2013), syntax abilities (Nauchi and Sakai, 2009; Pliatsikas et al., 2014) and executive language control (Elmer et al., 2011; Filippi et al., 2011; Abutalebi et al., 2012, 2013b; Zou et al., 2012). In addition to these studies on specific characteristics of L2 acquisition, research has also been devoted to overall second language proficiency (Amunts et al., 2004; Coggins et al., 2004; Mechelli et al., 2004; Osterhout et al., 2008; Mårtensson et al., 2012; Mohades et al., 2012; Ressel et al., 2012; Schlegel et al., 2012; Stein et al., 2012; García-Pentón et al., 2014; Klein et al., 2014), i.e., to a broad level of L2 proficiency as assessed by overall linguistic testing or by simply comparing groups of bilinguals to groups of monolinguals (Stein et al., 2014; Winkler, unpublished master thesis). Anatomical studies in bilinguals are based on the investigation of GM density changes (e.g., Mechelli et al., 2004; Osterhout et al., 2008; Stein et al., 2012), cortical thickness (e.g., Mårtensson et al., 2012; Klein et al., 2014), and GM volume (e.g., Golestani et al., 2007; Wong et al., 2008). In language-associated areas and, in particular, in areas implicated in control, these studies have generally found alterations due to L2 acquisition, that increase with growing L2 proficiency (for a review, see Li et al., 2014; Stein et al., 2014). Most recently, there have been morphometric studies suggesting that the consequences of bilingualism are related to the form of bilingualism, including not only the age of acquisition and proficiency, but also the context in which the two or more languages are used, i.e., whether speakers are immersed in the language environment, whether they learned the language in a classroom setting and so forth (see the current Special issue).

Neuroscientific studies with multilingual participants, i.e., with subjects speaking at least three languages fluently, are not carried out very often. Only recently, Abutalebi et al. (2013a) combined functional and structural MRI to examine the role of the basal ganglia in multilingual participants. Because of the permanent exposure to a major articulatory load when speaking several languages during a lifetime, the authors hypothesized that there is an enlarged density of gray matter in this area in multilinguals. Indeed, as compared to monolinguals, participants speaking three languages demonstrated increased GM density in the left putamen, which supports the notion of structural plasticity as result of handling a complex articulatory repertoire. In order to compare high cognitive, linguistic, and articulatory demands between multilinguals of two different sorts, namely professional multilingual interpreters versus control multilinguals, Elmer et al. (2014) conducted GM volume analysis on regions previously shown to support language control and executive functions in multilinguals. Interestingly, GM volume was found to be reduced in highly trained multilingual interpreters in a number of regions associated with language control, which suggests that intense training can result in more efficient neural networks, probably due to the pruning of superfluous connections.

Most of the research on the (co)-organization of several languages in the brains of multilinguals has been conducted by using functional brain imaging. Vingerhoets et al. (2003) demonstrated that – in multilinguals with comparable levels of proficiency – late L2 acquisition results in greater activation in L2 than in L1 (Vingerhoets et al., 2003; also shown by Perani et al., 2003; Wartenburger et al., 2003; Kovelman et al., 2008 in bilinguals). In quadrilingual subjects, although there was no clear association between the age of acquisition and the amount of activation, a negative correlation was found between the level of proficiency and the amount of activation (Briellmann et al., 2004). This was also suggested by the data of Abutalebi et al. (2013b) in trilinguals. Bloch et al. (2009) focused on the relation between the age of L2 acquisition and the variability of regional brain activation in Broca’s and Wernicke’s areas in subjects speaking at least three languages fluently and where the L3 had been learned after the age of 9 years. They demonstrated that variability in the representation of the three languages of the individual is related to the age of acquisition of L2, which indicates that early exposure to more than one language gives rise to a language processing network that can accommodate late learned languages.

The present study is based on the work of Bloch et al. (2009) and analyses structural MRI data of subjects fluent in at least three languages. The design of the study allowed us to search for structural differences between simultaneous and successive acquisition of L2. Thus, we suppose that simultaneous (SiM) versus successive or sequential acquisition of L1 and L2 (SuM) is associated with differences in the structural organization of brain areas subserving language processing. More precisely, we hypothesize that groups who acquired L2 later also show higher GM volumes in language-associated regions, as well as in other brain areas belonging to the extended language network (Ferstl et al., 2008). The design of the study provides us, further, with the opportunity to consider the potential role of the late L3 acquired by all participants. Structural differences between simultaneous (SiM) and successive multilinguals (SuMs) could indicate that very early acquired characteristics are maintained over a long period of life.

## Materials and Methods

### Subjects

Forty-four healthy, right-handed [verified by the outcome of the Edinburgh Handedness Inventory (Oldfield, 1971)], non-smoking multilinguals voluntarily participated in this study after receiving information about the investigation and the scanning process and giving their written informed consent. The subjects’ average age at MRI acquisition was 28 years (range 18–37 years) and their female/male ratio was 22/22. The Ethics Committee of the University Hospital of Basel (EKBB, Switzerland) approved the study and confirmed its compliance with all relevant regulatory standards. All subjects were fluent and of medium to high proficiency in at least three languages (see **Table 1**). They did not differ with respect to their acquisition of their L3, which was comparable within all groups, and acquired at 9 years of age or later at school (see Bloch et al., 2009).

**Table 1:** Language and proficiency profile of the 44 participants.

Group	Subgroup in Bloch et al. (2009)	L1		L2			L3	

			Level of competence		Level of competence	Level of immersion^1^		Level of competence
1 SiM	Simultaneous	English	*C2*	Swiss German	*C2*	High by context	Italian	*B*2
2 SiM	Simultaneous	Hungarian	*C2*	Swiss German	*C2*	High by context	English	*B*1
3 SiM	Simultaneous	Hungarian	*C1*	Swiss German	*C2*	High by context	English	*B*1
4 SiM	Simultaneous	Italian	*C1*	Swiss German	*B2+*	High by context	French	*B*1+
5 SiM	Simultaneous	French	*B2+*	Standard German	*C2*	High by context	English	*C*2
6 SiM	Simultaneous	French	*C2*	Standard German	*C*1	High by context	English	*B*2+
7 SiM	Simultaneous	Italian	*C1*	Standard German	*B2+*	High by context	French	*B*1+
8 SiM	Covert simultaneous	Italian	*C1*	Swiss German	*C2*	Medium-high by context	Spanish	*B*1+
9 SiM	Covert simultaneous	Italian	*C2*	Swiss German	*C2*	Medium-high by context	English	*B*2
10 SiM	Covert simultaneous	Greek	*B*1*+*	Swiss German	*C2*	Medium-high by context	Spanish	*C*1
11 SiM	Covert simultaneous	Slovene	*C2*	Swiss German	*C2*	Medium-high by context	English	*C*1
12 SiM	Covert simultaneous	French	*C2*	Standard German	*C2*	Medium-high by context	English	*B*2
13 SiM	Covert simultaneous	Serbo-Croatian	*C2*	Standard German	*C2*	Medium-high by context	English	*C*1
14 SiM	Covert simultaneous	Turkish	*C*1	Standard German	*C2*	Medium-high by context	English	*B*2+
15 SiM	Simultaneous	Standard German	*C2*	English	*B2*	High by family	French	*A*2
16 SiM	Simultaneous	Standard German	*C2*	Indonesian	*B1+*	High by family	English	*C*1
17 SiM	Simultaneous	Swiss German	*C2*	Italian	*C1*	High by family	English	*C*1+
18 SiM	Simultaneous	Swiss German	*C2*	Italian	*C2*	High by family	English	*C*2
19 SiM	Simultaneous	Spanish	*C2*	Catalan	*C2*	High by family	Swiss German	*C*2
20 SiM	Simultaneous	Spanish	*B2*	Catalan	*B1*	High by family	Standard German	*C*2
21 SiM	Simultaneous	Finish	*C2*	English	*C1*	High by family	Standard German	*C*2
22 SiM	Simultaneous	Portuguese	*B2*	French	*C1*		Japanese	*A*1
23 SiM^2^	Simultaneous	Catalan		Spanish			English	
24 SiM	Covert simultaneous	Bulgarian	*C2*	Russian	*C1*		French	*C*1

25 SuM	2nd to 5th year	Spanish	*C2*	Standard German	*C2*	High by context	English	*B*1+
26 SuM	2nd to 5th year	French	*B2*	Standard German	*C1*	High by context	English	*C*2
27 SuM	2nd to 5th year	Swiss German	*C2*	English	*C1*	Temporary high by context	French	*C*2
28 SuM	2nd to 5th year	Swiss German	*C2*	English	*C2*	Temporary high by context	Ivrit (New Hebrew)	*B*2+
29 SuM	2nd to 5th year	Swiss German	*C2*	English	*C*1	Temporary high by context	French	*B*1
30 SuM	2nd to 5th year	Standard German	*C2*	French	*C2*	Temporary high by context	English	*B*1+
31 SuM	2nd to 5th year	Standard German	*B2+*	French	*B2+*	Temporary high by context	English	*B*1+
32 SuM	2nd to 5th year	Spanish	*C*1	Italian	*B2*	Temporary high by context	Swiss German	*C*2
33 SuM	Late	Swiss German	*C2*	English	*C1*	Classroom learning	French	*C*1
34 SuM	Late	Standard German	*C2*	French	*B2+*	Classroom learning	Russian	*B*2+
35 SuM	Late	French	*C2*	Standard German	*B2+*	Classroom learning	English	*B*1+
36 SuM	Late	Swiss German	*C2*	English	*B2*	Classroom learning	French	*B*2
37 SuM	Late	Swiss German	*C2*	English	*C2*	Classroom learning	Italian	*B*2
38 SuM	Late	Swiss German	*C2*	English	*C2*	Classroom learning	French	*B*2+
39 SuM	Late	Italian	*C2*	Standard German	*B2*	Classroom learning	English	*B*2
40 SuM^2^	Late	Swiss German		French		Classroom learning	English	
41 SuM	Late	Swiss German	*C2*	French	*C2*	Classroom learning	English	*C*2
42 SuM	Late	Italian	*C2*	Standard German	*B2+*	Classroom learning	French	*B*2
43 SuM	Late	French	*C2*	English	*B2+*	Classroom learning	Standard German	*C*2
44 SuM	Late	Swiss German	*C2*	French	*B2*	Classroom learning	English	*B*2

*Here, the classification of the multilingual participants and their languages with individual proficiencies levels is presented. Multilinguals are grouped into SiM and SuM, depending on their age of L2 acquisition. Prototypical details on level of immersion of L2 are given. A1 and A2 refer to competence levels of the basic user, B1 and B2 to the independent user, and C1 and C2 to the proficient user. A2+, B2+, and C1+ are intermediate stages [Common European Reference Framework for languages (CERR), Council of Europe, 2001; North, 2000]. Given the diglossic situation in the German speaking part of Switzerland, Standard German, and Swiss German can both be regarded as varieties of German. ^1^The classification into different levels of immersion is qualitative–descriptive and not quantitative-categorical and is aimed to give further input on the characteristics of our individual subjects rather than to mark or define strict groups of learners. ^2^Subjects who did not return the CERR assessment form.*

### Assessment of Language Profiles

The multilinguals’ age of second language acquisition was defined after analyzing each individual’s language biography (Schütze, 1988; Schwabe, 2003) through oral interviews lasting 2–3 h. These in-depth linguistic biographies are based on the observation that free narrations give a more realistic account of the language history of the participant (Franceschini, 2002). The subjects could then be classified into four different groups of L2 acquisition: 16 (*F* = 10) simultaneous bilinguals, 8 (*F* = 3) covert simultaneous bilinguals, 8 (*F* = 3) sequential bilinguals, and 12 (*F* = 6) late multilinguals (Bloch et al., 2009). For the purpose of the present study, we re-grouped the participants into two groups:

(1) The *simultaneous multilingual* (SiM) group (*N* = 24; *F* = 13; average age: 27.7 years; age range: 18–36 years) consisting of simultaneous bilinguals, i.e., participants growing up in a bilingual family, where both parents/caregivers spoke different languages, and covert simultaneous bilinguals, i.e., participants growing up in a monolingual family whose language differed from that of the surroundings. This group of bilinguals was exposed to the L2 by the environment parallel to the L1, and for that reason they were grouped together with the simultaneous bilingual group.
(2) The *successive multilingual* (SuM) group (*N* = 20; *F* = 9; average age: 27.8 years; age range: 20–37 years) comprised successive bilinguals who had acquired their L2 subsequently to L1 between their second and fifth year of life, and late multilinguals who had acquired L2 at school when they were at least 9 years of age. Thus, this group covers multilinguals who acquired their L2 at a distinct time point *after* the acquisition of their L1.

**Table 1** shows information on the multilingual profiles of the SiM and SuM groups. Additionally, it gives information about the type of L2 immersion. The level of competence in the individual languages was scaled by self-assessment, using the Common European Reference Framework for Languages (CERR, North, 2000; Council of Europe, 2001). Calculations using the Mann–Whitney *U* Test revealed no significant differences between SiM and SuM concerning L2 and L3 proficiency (*p* > 0.05, one-tailed).

### Language Immersion Profile

As the age of acquisition is not the sole or exclusive influence to alter the structure of the brain, we provide here some additional, *post hoc* information on the *degree of immersion to L2* in our two groups of participants. Immersion has been shown to affect the brain’s anatomy (Pliatsikas et al., 2015) and can be defined as the amount of naturalistic exposure, or immersion, that the speakers receive to that language. It is the degree to which language learners are exposed in their day-to-day activities, (see Pliatsikas and Chondrogianni, 2015).

This study was conducted in the German speaking part of Switzerland. This resulted in the recruited participants having significant exposure to German (the exceptions are subjects 20, 21, 22, 23, and 24 of the SiM group who either acquired Standard German in classroom circumstances as L3 after the age of 9 years or who did not report speaking German at all or learned it as an L4; and subjects 35, 39 and 42 of the SuM group who acquired Standard German in classroom circumstances as L2, see **Table 1**). Growing up in a German-speaking country makes the context for the acquisition of the L2 – in the cases when Standard German/Swiss German was learned as an L2 – one of early and *high immersion by context* (due to the linguistic dominance of the environment), or, in the case of the covert simultaneous-participants, a context of *medium–high immersion*. Similarly, growing up in a German speaking country with at least one caregiver speaking another language than German makes L2 acquisition of *high immersion by family*. Thus the majority of the members of SiM acquired their L2 by high immersion by family/context or medium-to-high immersion by context. The majority of the members of the SuM group learned L2 in a classroom setting. The subgroup of multilinguals classified as “2nd to 5th year” of age spent a period of their childhood/youth outside a German speaking country or moved to a German-speaking context. Their level of immersion to L2 is thus either *high by context* (2 out of 8) or *temporary high by context* (6 out of 8). Therefore, the quality of immersion differs between SiM and SuM (**Table 1**).

### Magnetic Resonance Imaging

#### MR Image Acquisition

Magnetic resonance images of the 44 subjects were acquired on a 1.5-T Magnetom Vision MRI Scanner (Siemens, Erlangen, Germany) at the University Hospital of Basel. We used a standard head coil to restrict head movements and to limit motion artifacts. A three dimensional (3D) T_1_-weighted anatomical high-resolution Magnetization Prepared Rapid Gradient Echo (MPRAGE) sequence was applied with repetition time of 9.7 ms, echo time of 4 ms, inversion time of 300 ms, and isotopic spatial resolution 1 mm × 1 mm × 1 mm (see also Bloch et al., 2009). The scans were all screened for major radiological abnormalities or visual artifacts by an experienced neuroradiologist.

#### Voxel-Based Morphometry

These MRI data were analyzed on commercially available Intel-based desktop computers with a Debian Linux 3.1 operating system. The structural images were pre-processed using a Voxel-Based Morphometry (VBM8) toolbox^1^, as implemented in the Statistical Parametric Mapping software package^2^. The data were registered with “Diffeomorphic Anatomical Registration Through Exponentiated Lie” (DARTEL) within VBM8, running under the MATLAB 7.11.0 (R2010b) environment (Members of the Wellcome Trust Centre for Neuroimaging, 2009). Accuracy and sensitivity were maximized by creating a study-specific template and segmentation of each subject’s image (Yassa and Stark, 2009). We conducted the following steps: (1) checking for scanner artifacts and major anatomical abnormalities for each subject; (2) aligning and reorientating the scans; (3) using New Segmentation and high-dimensional normalization DARTEL (Ashburner, 2007); (4) checking for homogeneity across the sample; and (5) using 8 mm standard smoothing (Bailey, 2008). The default values for realignment, warping, and normalization were used (Kurth et al., 2010). Finally, realigned, segmented, normalized, and smoothed data were subjected to statistical analysis.

Of the 44 samples of this study two were outliers, with a mean covariance below 2 SDs. Repeated analyses without these two subjects did not change the results (not shown). We therefore decided to retain these two subjects.

### Statistics

Voxel-based morphometry compares images on a voxel basis after spatial normalization using deformation fields that discount macroscopic differences in shape. We estimated between-group differences in GM volume at each intracerebral voxel in standard space by fitting a full-factorial analysis of covariance (ANCOVA), and contrasted the SiM and SuM groups. We modeled age at image acquisition and sex/gender as covariates of no interest, in order to reduce the potential impact of these variables on the GM volume in language-associated brain areas. We identified spatially continuous voxels at a threshold of *p* < 0.01 (uncorrected; cluster forming threshold; Petersson et al., 1999) and defined a family wise error-corrected cluster-extent threshold of *p* < 0.05 to infer statistical significance. In order to mark areas with significant GM volume differences on this statistical threshold level, Montreal Neurological Institute (MNI) coordinates were transformed into Talairach space (MNI and Talairach Transformation, 2013; Talairach.org Daemon, 2013).

## Results

**FIGURE 1:**
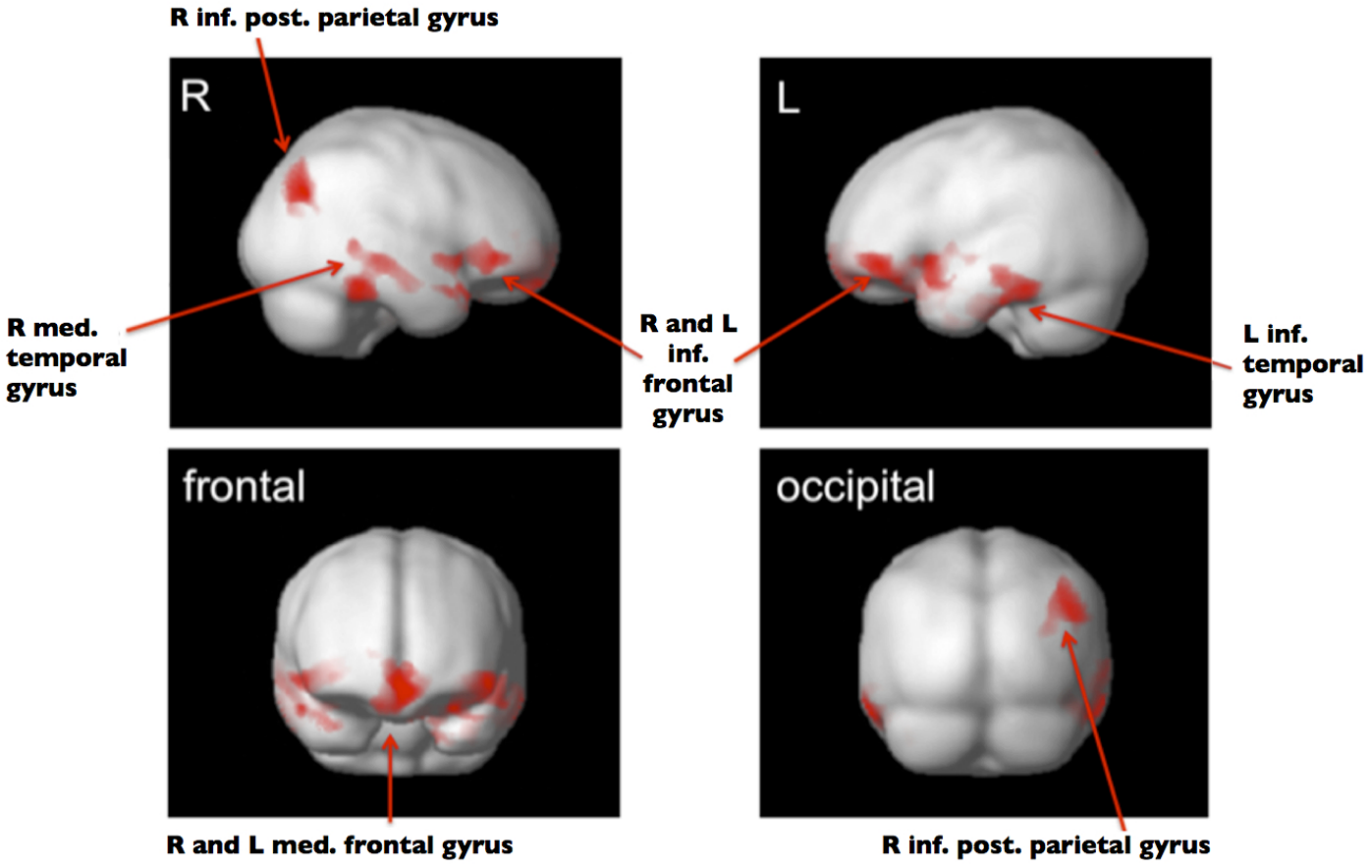
**ANCOVA full factorial 2 × 2**. Significant clusters (*P*_FWE-corr_ < 0.05) with smaller GMV in SiM versus SuM shown in red. Group template used as background image in gray.

Gray matter volume was lower in the group of SiMs as compared to SuMs in the following regions: bilaterally in the medial frontal gyrus (MFG) and the left inferior frontal gyrus (IFG; *p* < 0.001, FWE); in the right IFG and right medial temporal gyrus (MTG); in the left inferior temporal gyrus (ITG); and in the right inferior posterior parietal gyrus (*p* < 0.05, FWE; **Table 2**; **Figure 1**). The opposite contrast SuM > SiM did not reveal any significant results.

**Table 2:** Results SiM < SuM.

ANCOVA full factorial 2 × 2, SiM < SuM. Height threshold *p*_uncorr_ < 0.01; extent threshold *k* = 2497 voxels.					

Area	MNI coordinates of cluster maximum (x/y/z)	Cluster P_FWE-corr_	Cluster size>k_E_ (voxels)	Peak level T
Frontal lobe	Medial frontal gyrus	L R	−2/46/−9 2/56/−11	<0.001	4107	4.71
	Inferior frontal gyrus	L R	−38/36/−8 44/26/−2	<0.001 0.015	4753 2788	4.61 3.58
Temporal lobe	Inferior temporal gyrus	L	−63/−40/−24	0.016	2766	4.19
	Medial temporal gyrus	R	60/−27/−12	0.009	3071	3.90
Parietal lobe	Inferior posterior parietal gyrus	R	50/−79/48	0.027	2498	4.17

*Clusters with significantly smaller gray matter volume in SiM are displayed.*

## Discussion

The present study of 44 multilinguals is to our knowledge the first VBM study in multilinguals as opposed to numerous studies in bilinguals. It shows that GM volume was higher in the group of multilinguals who learned their L2 successively compared to the multilingual who acquired a second language simultaneously with their L1. Thus, subjects who did not acquire two languages simultaneously (by immersion) in early life but learned them sequentially, mostly in classroom settings, showed larger GM volume patterns in cortical language-associated regions and the extended language network (Ferstl et al., 2008). This result supports our thesis that early simultaneous bilingualism persists in the anatomical make-up of the adult brain.

The exact degree to which the difference between SiM and SuM is or is not influenced by a late learned L3 cannot be determined based on the present design. However, the other way round requires attention: testing trilinguals obliges us to tentatively hypothesize that despite the fact of a late learned L3 the differences based on the age of L2 acquisition persist into adulthood and do not disappear. Thus SiM and SuM remain different even though a late L3 was acquired. It can be assumed that the earlier in life a language experience is made, the more receptive the brain is to new learning and the more efficiently the brain can incorporate new language associated experiences, i.e., further input can be integrated into the same structural substrates. This would then be mirrored in GM patterns, particularly in the lower GM volume for early simultaneous L2 acquisition, including the late learned L3.

Our result corresponds with previous research showing an impact of L2 acquisition on GM volume of the bilingual brain (Mechelli et al., 2004; Osterhout et al., 2008; Stein et al., 2012) as well as research on GM volumetric changes in multilinguals (Elmer et al., 2014). However, the comparison of work applying GM density approaches and studies based on GM volumetric methods remains challenging. VBM is a technique that permits comparisons of the entire brain volume at the single voxel level. In contrast to previous studies (Mechelli et al., 2004; Osterhout et al., 2008; Stein et al., 2012) which reported GM densities rather than volumes, we used “optimized” VBM, which includes an additional modulation step to minimize the potentially confounding effects of errors in stereotactic normalization (Ashburner and Friston, 2001; Good et al., 2001). All images were smoothed using a 8 mm full-width-at-half-maximum Gaussian kernel, as in a previous study (Mechelli et al., 2004). According to the matched filter theorem, the width of the smoothing kernel determines the scale at which morphological changes are most sensitively detected (White et al., 2001). In the present study, we have chosen a rather small smoothing kernel, as this allows us to detect a greater number of regions with small structures and to better compare our results with the GM densities reported by Mechelli et al. (2004). Furthermore, both samples are based on healthy participants, so that we did not expect that regional changes would be very large or would differ much between cortical regions or between the studies. As expected, our results basically confirm the correlation (Mechelli et al., 2004; Stein et al., 2012) and/or association between age of L2 acquisition and GM structure, as reported previously (Osterhout et al., 2008).

In the present study, differences in GM volume were detected in the temporal as well as inferior and medial frontal regions of both hemispheres and the inferior parietal area. These are broadly parts of regions involved in the functional anatomy of language (Price, 2010) and may be linked, with the exception of the right inferior prefrontal cortex, to the “extended language network” outlined in influential work on the functional processing of language comprehension (Ferstl, 2007; Ferstl et al., 2008). Typically, primary language areas for language comprehension and production, such as Wernicke’s and Broca’s areas, did not display any significant difference between the SiM and SuM group. In the related fMRI study on multilingualism by Bloch et al. (2009), no specific group-dependent differences in activation were found in these areas either, which shows, for our population, that these regions are used, irrespectively of when a language is learned. The degree of variability in which they were activated by the three languages is, however, highly dependent on the age of L2 acquisition.

In the following, our GM data are discussed in relation to other studies on structural changes of gray matter linked to overall second language proficiency (Stein et al., 2014). Special attention is given to the bilateral character of our results. In their whole brain analysis of early and late bilinguals, Mechelli et al. (2004) found structural changes in the inferior parietal cortex (IPC) in relation to age; similarly Osterhout et al. (2008) employed an ROI analysis and detected structural alterations in the same region. Here too, GM changes in the IPC were detected and linked to age of acquisition. Others have demonstrated that the GM density of this region is positively correlated with vocabulary acquisition and knowledge, suggesting that this area is important not only for global L2 acquisition but for handling a large vocabulary (Lee et al., 2007; Richardson et al., 2010). Contrary to Mechelli et al. (2004), Osterhout et al. (2008), and Richardson et al. (2010), however, the present work showed changes in the right (x/y/z, 50/-79/48) and not in the left hemispheric IPC and thus supports the conclusions of Lee et al. (2007), who showed that vocabulary mastery predicted GM density in the bilateral IPC.

While Stein et al. (2012), in their longitudinal study on L2 acquisition, reported an increase in GM density in the left inferior frontal cortex (IFC) in close vicinity to the pars triangularis in Broca’s area, the present study detected a bilateral pattern in this region and revealed a difference with respect to the age of L2 acquisition: Stein et al.’s (2012) participants learned L2 as adults, whereas our group of participants acquired their L2 as children. The results in our group of SiMs in IFC is in line with data of a recent cortical thickness study concerning the bilaterality of the structural patterns in this very same region (Klein et al., 2014). Klein et al. (2014) report, however, that thickness correlates positively with age of acquisition in the left IFG and negatively in the right IFG; this opposing interhemispheric effect was not re-enacted in our study where the association of age with higher GM volumes clearly counts for both the right and left IFG.

Our results on bilateral differences between SiM and SuM in GM volume in IFC are corroborated by functional data from bi- and multilinguals showing greater bilateral activation during both L1 and L2 processing than for monolinguals (Hull and Vaid, 2006; Park et al., 2012), with a strong tendency for the right Broca’s homolog to be activated in the L1 of multilinguals (Kaiser et al., 2007). There is still little evidence about the function of the MFG in the context of structural changes due to L2 acquisition, except that its cortical thickness shows alterations after very intensive language training over many months (Mårtensson et al., 2012). Again, our present results reveal structural bilateral modifications in the MFG, whereas Mårtensson et al. (2012) found these only in the left side. Data based on functional MRI studies suggest that the MFG is crucial for text comprehension (Ferstl and von Cramon, 2001).

It is well known that both the inferior and the middle temporal gyrus handle various aspects of lexical semantic representation and processing. Our study is, to our knowledge, the first to present changes in the GM volume due to early L2 acquisition in both the right MTG and left ITG.

Taken together, the right hemispheric trend (as exemplified in r-IPC; bilateral IFC; bilateral MFG; r-MTG) – which characterizes our set of multilinguals when differentiated by the age of L2 acquisition – could be influenced by two additional factors: by the mode of L2 acquisition and/or by the interference of the L3.

Our subjects were carefully selected on the basis of their multilingual profile and had to undergo extensive interviewing for 2–3 h about their three languages. However, some information about their languages was not captured. Thus, for instance, it cannot be excluded with certainty whether any of the simultaneous participants registered with German/Swiss German as L2 grew up in a non-German speaking country and acquired L1 from one caregiver and L2 (German/Swiss German) from a second caregiver. Nevertheless, the presence of immersion as the main access to L2 acquisition remains valid in SiM, as well as the presence of classroom learning in SuM. Future cross-sectional and longitudinal research is needed to identify which of these ways of learning L2, i.e., L2 acquisition based on *high immersion by family, high immersion by context, medium-high immersion by context, temporary high immersion by context*, or *classroom learning* has a greater impact on structural GM in relevant brain areas. Most recently, Pliatsikas et al. (2015) demonstrated the effects of immersion on brain structure in young, highly immersed late bilinguals. In their view, “[immersion] can be broadly defined as the degree to which language learners use their non-native language outside the classroom and for their day-to-day activities and usually presupposes that the learners live in an environment where their non-native language is exclusively or mostly used” (see Pliatsikas and Chondrogianni, 2015). Interestingly, structural alterations in white matter were shown to be effected by everyday L2 use in a naturalistic environment, rather than by length of L2 learning or age of onset of L2 learning (Pliatsikas et al., 2015). Thus, our results in the group of SiM can also be interpreted from this perspective showing that naturalistic exposure, rather than age of L2 acquisition, impacts on brain structure. This is not the case for the group of SuM who in their majority acquired L2 as a foreign language in the classroom.

Finally, the impact of the L3 on language-associated brain areas and the extended language network remains to be elucidated. In our experimental setup, the age of L2 acquisition is the variable determining the GM structure early in development. However, the way in which training for additional languages drives GM plasticity in regions already influenced by bilingualism is open to speculation. Additional training might result in pruning of language networks, as suggested by Elmer et al. (2014), or might drive contralateral (right hemisphere) cortical areas to participate in language related tasks.

The results of the present study cannot prove a lifelong plasticity of the brain for languages since the examined subjects were chosen based on the fact of having learnt at least three languages during early or late childhood, respectively, and we do not know about further changes in their brains during adulthood. Neither do the obtained outcomes give any information if there is a critical age for the native-like acquisition of one or many languages, although there are certainly studies showing complex relations between the maturation of the brain in children and the brain’s plasticity to adjust to structural demands of (individual) language development (Brauer et al., 2011).

### Methodical Considerations

Modification of the user-options implemented in the analysis software, such as setting the smoothing kernel, can influence subsequent statistical results (Ashburner and Friston, 2001): a larger kernel (12 mm instead of 8 mm) results in greater cluster sizes (Friston, 2003, p. 5). We used a default option of 8 mm as recommended. VBM compares voxel-by-voxel images of different groups and reports MNI standard coordinates for every cluster center, which does not necessarily correspond to the actual cluster localisation in an individual’s brain. Thus VBM statistics do not differentiate between two clusters localized for example in the medial plane. In the present case, the large clusters in the medial frontal gyri on the left and the right are counted as one cluster.

As to the statistical thresholds, it is still very difficult to compare data from different studies. Height thresholds range from *p*_uncorr_ < 0.001 up to *p*_corr_ < 0.05 in different studies (Brambati et al., 2004; Eckert et al., 2005; Silani et al., 2005; Hoeft et al., 2007; Steinbrink et al., 2008; Richardson and Price, 2009). Here, an uncorrected height threshold of *p* < 0.01 was used. The clusters found are therefore large, although there are small differences between the two groups.

## Conclusion

Contrary to the successive acquisition of the second language, simultaneous acquisition of L1/L2 (by immersion) from the first year of life on is associated with low GM volume in language-associated regions, in the prefrontal, medial temporal and parietal cortex, in particular. This difference persists even though a late L3 is learned. Growing up in a multilingual environment in early childhood may change the individual’s cortical structure, enforcing it to generally build more efficient synaptic networks for language processing. To further understand structural changes underlying brain plasticity during language learning requires longitudinal studies with homogenous groups of SiM and SuM.

## Conflict of Interest Statement

The authors declare that the research was conducted in the absence of any commercial or financial relationships that could be construed as a potential conflict of interest.

## Footnotes

1 http://dbm.neuro.uni-jena.de/vbm8/

2 http://www.fil.ion.ucl.ac.uk/spm/software/spm8/
